# Gingivitis and Its Relation to Crown Work

**Published:** 1898-07

**Authors:** S. B. Palmer

**Affiliations:** Syracuse, N. Y.


ARTICLE IX.

### GINGIVITIS AND ITS RELATION TO CROWN WORK.

S. B. PALMER, M. D. S., SYRACUSE, N. Y.

Animal magnetism is not a new term, nor does it need evidences of its existence. Animal heat is also a fixed fact; and is one of the best proofs of organization to meet the conditions. Animal heat ranges between freezing 32° and 100¾° above when the organized dynamo burns out and vital energy ceases.

Before making an application of these organized forces please bear in mind that electricity and heat are close friends helping each other in most of the work done by those forces. The effect of this union upon gum tissue is this : Gold in the mouth will not injure gum tissue by the temperature, conducted by hot drinks or food as all know who wear or have worn gold plates; but where gold is insulated from the membrane, except at a given point or points, there is added to heat a current of electricity which, with the heat, is discharged into the gums, etc.

Mention will be made of this fact further on. Now let us introduce quotations from the article already mentioned.

"It is certainly a lamentable fact to note the surprisingly small percentage of roots carrying crowns that are devoid of any evidence of periosteal Inflammation, or that are surrounded by tissue presenting a normal or healthy appearance as compared with the very great percentage that invariably manifest more or less marked degrees of gingivitis."

On the face of the great amount of literature that has been given us upon this subject, and in view of the rapid strides of progress and advancement achieved in this line, it seems indeed a deplorable condition when one can conscientiously and candidly assert that only from 15 to 25 per cent. would be a fair estimate of these roots, which after carrying crowns for a time, present no indications of this condition, and yet we are convinced by close observation that on an average such is the case. The question naturally arises then as to its probable cause.

Since a very great percentage of the crowns now in use are made of gold, or having gold bands, would it be just to attribute it to the assertion sometimes made that the tissues of the mouth take unkindly to that metal, when we will, perhaps, turn right around and advocate a gold plate to the next patient presenting themselves; when we know as an absolute certainty, vouchsafed to by proof and experience, that there is no substance, either metallic or mineral, that when brought into contact with the tissues of the mouth presents so many favorable points as gold. Yet, for all that, in crown work we can but acknowledge the frequent presence of an unfavorable condition, but does it not seem very plausible that the fault lies not in the metal, but in its unnatural relation to the root and tissue by virtue of poor adaptation? Please bear this in mind, it will be answered farther on.

"It is an acknowledged fact that the tissues take very kindly to porcelain, and that a much greater proportion of crowns made of that material are worn with comfort and without the presence of inflammation and subsequent secession than are those made of gold; but it looks probable, and in fact seems evident to me, that that difference is due in main to the fact that a porcelain crown, when adapted and in position, presents always this favorabie condition, a perfectly smooth vitrified, highly-polished surface, with a rounding edge. And as this is the most desirable and natural condition why then would not the tissues take kindly to it and remain normal and healthy? When, unless there is irritation from an improperly prepared root, a poor fitted band, or impingment upon the membranes caused by driving the band on too far there is absolutely nothing to prevent. We are impelled to maintain, without hesitancy, that this can be accomplished, and the same results secured in the use of a gold crown if we will but take the time, pains and precaution to properly prepare our roots," etc. The remainder of the article describes how it may be done.

In all my reading I have not had such an opportunity to explain the cause of crown work disturbances, as the above affords. The differences between gold plates and gold crown, between porcelain and gold banded crowns, the incompatibility of gold as a filling material for specific conditions of teeth, may all be answered under one head: The effect of gold crown upon gum tissue.

Early prejudices against the electro-chemical theory seems to have excluded it from our dental chemistry, and with few exceptions, from being taught in dental colleges. As this journal reaches many readers who may not know the source from which the basal principles of our knowledge has been obtained we will repeat in substance what has several times been given in previous papers. In boyhood days fondness for the study of electro-chemistry by private study afforded considerable information.

In 1847 I commenced the study of dentistry, at the same time the wearing of a silver plate. Rubber was not then introduced. With that I commenced the study of oral electricity.

During fifty years a number of plates of various materials have been worn, each giving experience that could not have been gained by any other means. This information is of no account except as it may benefit some young practitioner by knowing points regarding metals worn in the mouth which are not recorded. I will briefly mention the peculiarities of each in turn. In early times silver was used for temporary work, also when gold could not be afforded.

Silver in the mouth is a positive element, corresponding to the zinc element in a galvanic cell, consequently any food containing carbon, as toast, broiled meats, roasted pea-nuts, coffee, etc., produced an unpleasant metallic taste, sulphur in eggs, breathing smoke from gunpowder, or even passing a sulphur spring, in breathing through the mouth the taste would detect sulphur. Still another feature, gold clasps were often used on silver plates, destruction of the teeth soon followed. The clasps, being negative, became the positive pole from which the current went out to decompose the impacted food, and thus furnish acid to dissolve the enamel. A gold plate, with an alleged silver clasp, would be an improvement, or a silver plate with an alloy of silver and copper clasps would have been a great improvement. The silver plate was discarded for gold, which was a relief from the taste of a metal. With gold the current was reversed, the plate was negative, and not acted upon by food like carbon, sulphur, etc.

It was a galvanometer, however, to detect base metals or metallic compounds. Silver, iron, tin, lead or liquids such as we often use in canned fruits, made it known. Thus the study became exceedingly interesting. Rubber came and was put to the test, and the result may surprise those who have not made the change. It is true patients could not describe if they could realize what an expert could detect. Rubber was an insulator, and here let me caution any young dentist against arguing that rubber plates do not injure taste. It may be true that the plate does not cover membranes of taste like the tongue, but the roof of the mouth has its office to perform on this line. Insulation was most objectionable, and that means more than can be imagined.

Thermal changes during eating are of importance and natural. The portion covered by a rubber plate might as well be paralyzed as to be so covered. The smooth surface of a plate of any material is unnatural, but when no changes, electrical or caloric are felt, taste is impaired, besides the abnormal condition of the surface covered, which I have not been able to correct by black rubber.

Please understand that I am not advocating disuse of rubber. If I was working at prosthetic dentistry I would use rubber according to circumstances. It does no material injury, but is annoying, as above stated, and I am giving the facts relative to each material.

Swaged aluminum plate teeth, mounted with rubber, was the next on trial, and at once overcame all objections. To say nothing of its light weight, the metal seldom produces electro-chemical action by anything taken into the mouth. It occupies a neutral position, as compared with gold or silver. The latter is a positive element, and gives a metallic taste, as before stated, by carbon, sulphur, etc., while gold is a negative factor, and consequently it becomes a receiver of the electricity which is generated during mastication, and mingling of the positive and negative elements of food. Electricity is thus generated, as it would be in mixing elements in the laboratory. Without a metal in the mouth the electricity is what might be called organic, not polarized. It is the agent which adds delicacy to taste, also that which prompts natural selection of food to be eaten in pairs, as sweet and sour, in lemonade, acid and alkali, soda water, charged waters, roasted or broiled meats, roasted coffee, peanuts, etc., are negatives to saliva, and cause a natural current of animal electricity. This prepared food is taken into the stomach and the mysterious process of digestion completed, neutralization of the elements, the waste product is cast off, the electric vital energy is stored up in the muscles for work. Excuse this digression from the oral cavity. The truth of this will some day revolutionize physiology and establish digestion upon an electrical basis.

Now we are ready to advance another declaration. That metal in the mouth converts organized electricity into physical electricity. The effect is this, when such an unnatural current is discharged at a given point tissue or dentine is injured. Albuminum is almost free from potential, thermal changes and electricity passes readily through the plate without injury, so with gold when it rests upon membrane. This will be taken up again.

The next trial plate was of cast aluminum for experimental purposes. In order to cast aluminum it is slightly alloyed, and my first experience in wearing the plate was interesting and amusing. The plate was a full upper, except the second molor on each side. To increase the bite, and to prevent the molars from elongating a cap of the metal covered the coronal surface. The case articulated with a bridge on each side below. The plate was inserted in the afternoon, nothing remarkable was noticed except the metallic taste thought to be due to finish, etc. In the evening I went upon the street, and to my surprise all the street lights in sight flashed, and were unsteady. As all were affected alike I concluded the cause was at the Central Station. It was not many minutes before I could produce the flickering at will by closing my teeth. On opening the jaws enough to give space the lights were steady, By involuntary action the electric shock affected the optic nerve and produced the effect. The gold became charged, and on contact discharged and gave a shock. Burring out the metal and filling it with vulcanite corrected the trouble. But the cast metal was discarded for a rolled and swaged plate, which, to me or for me, is the most comfortable plate I have worn, and, in fact, it has very few objections.

To be fair with the cast metal I will say for a single plate, where there would be no gold opposite, I presume the alloy would not be objectionable. In my own case, with a large surface of crowns below, and the tongue connecting the two metals, a metallic taste was ever present.

Now let us return to the subject of gingivitis. The cause of gold crown disturbance has been well defined in the effects of gold upon tissue we will recapitulate. Gold in the mouth becomes charged with electricity when the metal rests upon gum tissue; like a plate no harm is done when only small portions rests upon the gums or under the gums that portion receives the amount of heat and electricity that is received upon the whole surface. I know from experience that hot water alone produces severe pain on the sensitive dentine below the gum line. That coffee increases the current from the effects of the carbon in the coffee acting upon the saliva. I find that bridges are worse than single crowns, having more gold surface to gather thc electricity. Bear in mind that in physics intense heat can be produced by electricity. In animal life the range is between freezing, and 100.3—4° above. Cataphoresis teaches that intense currents destroy gum tissue; feeble currents, for a long period, produce like effects on organized bodies. It is a natural law that gold terminating in a band under the gum causes an abnormal condition as seen in connection with crown work. Perfect in fitting can not set this law aside. As a remedy for sensitiveness of dentine silver nitrate is the most effectual I have used. Shortening the band, so as not to extend beneath the gum, is usually a remedy for inflammation.

With the above explanation, aided by evolution in practice, with a better general understanding of oral electricity, I will try to make plain why gold fillings are not compatible with dentine in teeth of children, or in deep-seated cavities where the tissue is a conductor, without an insulating lining. As above stated, gold worn in the mouth becomes the positive plate or pole of a battery. During mastication or mingling of positive and negative elements with saliva, gold crowns are highly charged and the current passes into the gum tissue when the band extends under the gums. That is, the gold in contact becomes an electrode. To carry out the correspondence, every gold filling at a point nearest the pulp, or any part of the walls of the cavity which may be the best conductor, is an electrode. Where the dentine is normal, or when the dentine has been protected so as to insulate the current, no harm follows. Where the current continues, dissolution of the lime salt is the result, though it may be years in doing its work. To relieve pulp irritation caused from thermal changes, I have removed large gold fillings which were done by different operators, and had given no trouble, nor did they show indication of leakage, and found the dentine covering the pulp decalcified a nice organic conductor. The same principle is active when children's teeth are filled before becoming dense enough to insulate the thermal changes; in this case all of the cavity walls are softened. This doctrine has been taught for a score of years or more and has had but feeble support, having been declared by the highest recognized leaders in the profession that the so-called "electro chemical theory has no scientific foundation." In charity I forgive them, "for they know not what they do." I earnestly invite scientific discussion upon the laws set forth. I fully understand why a professor in physics should oppose the idea of evolution in physical laws. It is difficult to conceive why a dentist who has a knowledge of electro-chemistry cannot see that the same laws act and react upon organized matter, since he is permitted to work in a vital laboratory where the evolution is demonstrated and one is permitted to come in touch with nature and witness the evolution of matter to become organic, and, not less, the evolution of laws and forces to correspond with the material changes. My observations and personal experiences on this plane are backed by nature, and here I have the courage of my convictions to stand against the world's opposition from the plane below.—*Dominion Dental Journal.*

